# Long-term kidney outcomes in survivors of Wilms tumor: a single-center retrospective cohort study

**DOI:** 10.1007/s00467-024-06624-x

**Published:** 2025-01-09

**Authors:** Shannon Reinert, Stefanie W. Benoit, Rajaram Nagarajan

**Affiliations:** 1 Department of Nephrology, Cincinnati Children’s Hospital Medical Center, Cincinnati, OH USA; 2 Cancer and Blood Diseases Institute, Cincinnati Children’s Hospital Medical Center, Cincinnati, OH USA; 3 Department of Pediatrics, University of Cincinnati College of Medicine, Cincinnati, OH USA; 4 Department of Pediatrics, Texas Children’s Hospital, Baylor College of Medicine, Houston, TX USA

**Keywords:** Wilms tumor, Late effects of cancer treatment, Cancer survivorship, Pediatric cancer

## Abstract

**Background:**

Several studies have investigated long-term kidney outcomes in survivors of Wilms tumor (WT). However, many have small sample sizes, and there is a wide variation in reported outcomes. The aim of this study is to investigate the long-term kidney outcomes in survivors of WT (S-WT), including those patients considered to be at high risk for poor kidney outcomes, and using updated estimated glomerular filtration rate (eGFR) equations.

**Methods:**

This was a retrospective chart review of 64 patients treated for WT at a single pediatric center. Patients were off treatment for 5 years or more at the time of analysis and were evaluated for decreased kidney function, hypertension, proteinuria, and compensatory hypertrophy of the contralateral kidney.

**Results:**

At a median follow-up time of 11.3 years off treatment (range 5–22.6) and average age of 16.7 years (range 6.5–30), 35 patients had a decreased eGFR (< 90 mL/min/1.73 m^2^), and 2 patients had progressed to chronic kidney disease stage 5. Compensatory hypertrophy was observed in 67% of cases. 41% of patients had elevated clinic blood pressures, with 2 patients on an anti-hypertensive medication. Three of 9 patients had evidence of hypertension on ambulatory blood pressure monitoring. Eight of 37 patients (22%) had proteinuria.

**Conclusions:**

Kidney dysfunction is common in S-WT at a young age. This population should be carefully monitored for the development of decreased eGFR, hypertension, and proteinuria as part of their routine survivorship care. This is particularly true for modifiable risk factors of chronic kidney disease progression, such as hypertension and proteinuria.

**Graphical Abstract:**

**Figure:**
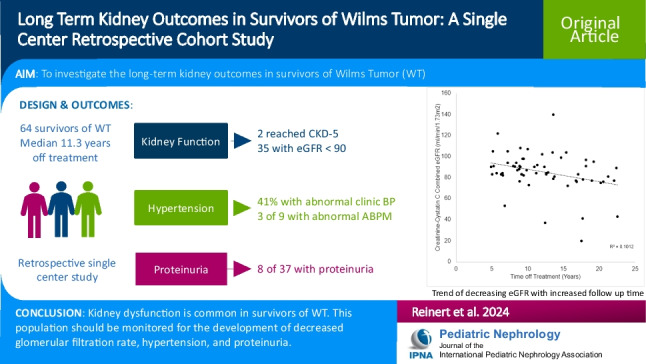
A higher resolution version of the Graphical abstract is available as Supplementary information

**Supplementary Information:**

The online version contains supplementary material available at 10.1007/s00467-024-06624-x.

## Introduction

Wilms tumor (WT) is the most common pediatric kidney tumor, affecting roughly one in 10,000 children [1]. Compared with other pediatric malignancies, WT has an excellent prognosis. As a result of randomized clinical trials and improvements in treatment protocols, WT outcomes have significantly improved over the past few decades, and studies now report overall 5-year survival rates of over 90% [2]. Treatment of WT is achieved with a multimodal approach that includes partial or radical nephrectomy, chemotherapy, and in some cases, radiation. With improved survival rates, long-term adverse therapy effects of WT treatment are even more important to understand. Given that most survivors of WT (S-WT) have undergone nephrectomy and have only a solitary functioning kidney [1, 3], it is important to understand the long-term kidney outcomes in this population.

There have been several studies evaluating long-term kidney outcomes in S-WT, including decreased glomerular filtration rate (GFR), proteinuria, lack of compensatory hypertrophy (CH) in the contralateral kidney, and hypertension. However, many of these studies have small sample sizes, and there is a wide variation in reported outcomes. The percentage of S-WT with decreased GFR has varied from 0 to 56% of patients [4]. Additionally, serum creatinine is the only biomarker used in most prior publications. Although creatinine is the most widely used endogenous marker to predict GFR, it has its limitations, including the fact that it is significantly affected by muscle mass [5]. Recent studies have demonstrated that S-WT are at risk for decreased BMI [6], decreased lean body mass [7], and sarcopenia [8], particularly after receiving radiation to the abdomen. Thus, creatinine may not be a reliable marker of kidney function in this population. Cystatin C (cysC) is an alternative biomarker that has been shown to be a strong predictor of kidney function, particularly in the pediatric population [5, 9, 10]. Combining both biomarkers in new, combined estimated GFR (eGFR) equations detects chronic kidney disease (CKD) in children more accurately [11, 12]. With regard to hypertension, in the largest follow-up study consisting of 1171 S-WT, 7% of patients had hypertension at 5 years [13]. Smaller studies have reported the prevalence of hypertension to be 0–20%. Proteinuria, a known risk factor for chronic kidney disease, has been seen in 5–57% of patients who survived WT [4]. Finally, a lack of CH has been seen in 3–45% of this population [4].

In addition to the wide variability in published outcomes, many studies have excluded those patients who are presumably at the highest risk for poor kidney outcomes. Green et al., Kostel et al., and Interiano et al. investigated long-term kidney outcomes only in patients with unilateral, non-syndromic WT [14–16]. Green et al. also excluded any patients with surgery on the contralateral kidney, tumor progression or recurrence, exposure to nephrotoxic chemotherapy (cisplatin, carboplatin, or ifosfamide), or radiation to the lungs. Interiano et al. excluded any patients treated with nephrotoxic chemotherapy or radiation. One larger study (61 patients) evaluated long-term kidney outcomes in S-WT, including patients with bilateral disease as well as those with an accompanying syndrome, and found 62% of patients to have at least one kidney sequelae, with 39% having decreased eGFR [17]. This is despite the fact that no patients in their study received whole abdominal radiation.

The aim of this study was to investigate the long-term kidney outcomes in all S-WT at our institution, using updated combination GFR estimation equations and including those with risk factors that put them at higher risk for kidney dysfunction.

## Patients and methods

### Patients

We performed a retrospective analysis of patients treated for WT at Cincinnati Children’s Hospital Medical Center (CCHMC) who were diagnosed from March 2000 to March 2018. Study patients were identified using the CCHMC Cancer Survivorship Database. Eligibility for this study included the following: (1) diagnosis of WT, and (2) follow-up in CCHMC Cancer Survivorship Center for at least 1 visit. The Cancer Survivorship Center is a long-term follow-up clinic for patients who are ≥ 5 years off therapy and in remission. Sixty-four patients were identified, and their medical records were reviewed.

Patient demographics (age, sex, race), tumor demographic (affected kidney(s), tumor stage), treatment details (chemotherapy regimen, radiotherapy regimen, type of surgery), and anthropometrics (height and weight) were abstracted by medical record review. Institutional Review Board approval at Cincinnati Children’s Hospital was obtained for this study (approval no. 2021–0421).

### Investigation of kidney outcomes

To evaluate for evidence of hypertension (HTN), proteinuria, and decreased kidney function, we abstracted information regarding serum creatinine, cysC, and blood pressure (BP) at 5-year intervals following completion of treatment, and yearly intervals for the past 5 years. GFR was estimated using the CKiD U25 equations based on serum creatinine and cysC [11, 12]. When cysC was available, cysC-creatinine-combined eGFR was used for analysis; otherwise, creatinine-only eGFR was used. CysC-only eGFR data are also provided for comparison. Pre-IFCC cysC results were multiplied by 1.24, prior to input into the U25 estimation, which is based on IFCC-calibrated serum cysC levels [18, 19]. Evidence of proteinuria was defined as a urine protein-to-creatinine (UPC) ratio > 0.2 mg/mg at any follow-up visit after the completion of treatment. Hypertension was assessed by office measurements of BP at nephrology and cancer survivorship clinic visits. Elevated BP and HTN were diagnosed based on 2017 AAP guidelines (age < 18 years old) and 2017 AHA guidelines (age ≥ 18 years old). Abnormal casual BP was defined as elevated, stage 1 HTN, or stage 2 HTN at 2 out of 3 of the last 3 measurements. When available, ambulatory blood pressure monitoring (ABPM) data were also extracted. Ambulatory BP measurements were classified as normal, sustained HTN (daytime and nighttime HTN), isolated daytime HTN (daytime hypertension with normal BP overnight), isolated nighttime HTN (nocturnal HTN with normal BP during the daytime, IHN), and masked HTN (normal office BP with abnormal ABPM results). A non-dipping pattern was defined as a decline in average systolic or diastolic BP of less than 10% at night. We collected the contralateral kidney length (cm) in all ultrasound reports available in our records. Contralateral CH was defined as 2 standard deviations above the mean kidney length based on height [20]. When height at the time of ultrasound was not available, kidney hypertrophy was defined as 2 standard deviations above the mean kidney length based on age and sex. The Childhood Cancer Survivor Study (CCSS) kidney failure model was used to predict the risk of late-onset kidney failure by age 40 [21]. The CCSS kidney failure model classifies 5-year survivors of childhood cancers as low, moderate, and high risk of late-onset kidney failure using demographic and treatment characteristics. Characteristics associated with a higher risk included race/ethnicity (Black, non-Hispanic), less than 10 years of age at diagnosis, nephrectomy, nephrotoxic chemotherapy (ifosfamide and platinum agents), cardiotoxic chemotherapy (anthracycline), abdominal radiation with mean kidney radiation dose 12 + Gy, GU anomalies, and hypertension within 5 years of diagnosis.

### Statistical analysis

Descriptive statistics included median values and interquartile ranges (IQR) or frequencies and proportions as appropriate. Distributional differences between groups were analyzed with the Chi-Square test. Given the limited sample size, a multivariable regression analysis was not performed. All tests were two-tailed with *P* < 0.05 considered statistically significant.

## Results

### Patient and disease characteristics

A total of 64 patients were included in the study. Patient characteristics are summarized in Table 1. Thirty-three patients (52%) were male. Nine patients self-identified as African American (AA), and 2 patients self-identified as Hispanic. The median age at diagnosis was 3.25 years (range, 0 to 8.6 years), and the median follow-up time was 11.25 years (range, 5 to 22.6 years). Four patients had conditions with known WT associations (mosaic trisomy 18 [22], isolated hemihyperplasia [23], REST gene mutation [24], and Beckwith-Wiedemann Syndrome [25]). Two patients had syndromes with unknown associations with WT (22q11 deletion syndrome and Pierpont syndrome). No patients had genitourinary abnormalities.

**Table 1:** Clinical and demographic characteristics of 64 Wilms tumor survivors

		Total	eGFR > 90 (ml/min/1.73 m^2^)	eGFR < 90 (ml/min/1.73 m^2^)
Total subjects (M/F)		64 (33/31)	29 (45%)	35 (55%)
Median age at diagnosis, *years* (IQR)		**3.25 (1.67–4.91)**	3.33 (1.67–4.83)	3.17 (1.67–5.17)
Race, *n* (%)	White	**52 (81%)**	23 (44%)	29 (56%)
	Black	**10 (16%)**	4 (40%)	6 (60%)
	Other	**1 (2%)**	1 (100%)	0
	Unknown	**1 (2%)**	1 (100%)	0
Ethnicity, *n* (%)	Non-Hispanic	**62 (97%)**	27 (44%)	35 (56%)
	Hispanic	**2 (3%)**	2 (100%)	0
Wilms tumor stage, *n* (%)	Stage 1	**8 (13%)**	4 (50%)	4 (50%)
	Stage 2	**21 (33%)**	7 (33%)	14 (67%)
	Stage 3	**17 (27%)**	11 (65%)	6 (35%)
	Stage 4	**12 (19%)**	4 (33%)	8 (67%)
	Stage 5	**6 (9%)**	3 (50%)	3 (50%)
Tumor localization, *n* (%)	Left	**30 (47%)**	13 (43%)	17 (57%)
	Right	**28 (44%)**	13 (46%)	15 (54%)
	Bilateral	**6 (9%)**	3 (50%)	3 (50%)
Relapse, *n* (%)		**3 (5%)**	1 (33%)	2 (67%)
Surgical procedure	Radical nephrectomy	**61 (95%)**	27 (44%)	34 (56%)
	Partial nephrectomy	**3 (5%)**	2 (67%)	1 (33%)
Type of chemotherapy	Vincristine	**64 (100%)**	29 (45%)	35 (55%)
	Dactinomycin	**63 (98%)**	28 (44%)	35 (56%)
	Doxorubicin	**36 (56%)**	20 (56%)	16 (44%)
	Cyclophosphamide	**13 (20%)**	7 (54%)	6 (46%)
	Etoposide	**13 (20%)**	7 (54%)	6 (46%)
	Carboplatin	**3 (5%)**	2 (67%)	1 (33%)
Radiotherapy	Any radiotherapy, *n* (%)	**30 (47%)**	16 (53%)	14 (47%)
	WART, *n* (%)	**11 (17%)**	6 (55%)	5 (45%)
BP follow-up time, *years*, *n* (%)	5–9	**25 (39%)**		
	10–14	**19 (30%)**		
	15–20	**12 (19%)**		
	20–22	**8 (12%)**		
eGFR follow-up time, *years*, *n* (%)	5–9	**28 (44%)**		
	10–14	**18 (28%)**		
	15–19	**12 (19%)**		
	20–23	**6 (9%)**		

*M*, male; *F*, female; *IQR*, interquartile range; *BP*, blood pressure; *eGFR*, estimated glomerular filtration rate

Creatinine-cystatin C–combined eGFR used when available, otherwise based on creatinine-only eGFR

Patient disease characteristics are summarized in Table 1. Eight patients (13%) had stage I disease by the Children’s Oncology Group staging system, 21 patients (33%) had stage II, 17 patients (27%) had stage III, 12 patients (19%) had stage IV, and 6 patients (9%) had bilateral disease (systemic stage V) at diagnosis. Surgical interventions included partial nephrectomy (2 patients), bilateral partial nephrectomy (1), and radical nephrectomy of the affected kidney (61). One patient required a bilateral nephrectomy due to the extent of the disease, followed by dialysis, and ultimately a kidney transplant. One of the patients who initially underwent a radical nephrectomy of the affected kidney later relapsed in the remaining kidney, requiring a partial nephrectomy of the remaining kidney. All patients received chemotherapy with a combination of two to six agents (vincristine, ± dactinomycin, ± doxorubicin, ± cyclophosphamide, ± etoposide, ± carboplatin). Three patients (5%) received a nephrotoxic chemotherapy agent (carboplatin). Twenty-five patients (39%) received radiotherapy. In 11 of these 25 patients, the unaffected kidney was in the radiotherapy field (whole abdominal radiation), with radiation doses ranging from 1050 to 2100 cGy (mean 1153 cGy). Thirty-two patients (50%) had previously had a visit with nephrology, and 25 (39%) had seen nephrology more than one time.

### Glomerular filtration rate

**Fig. 1:**
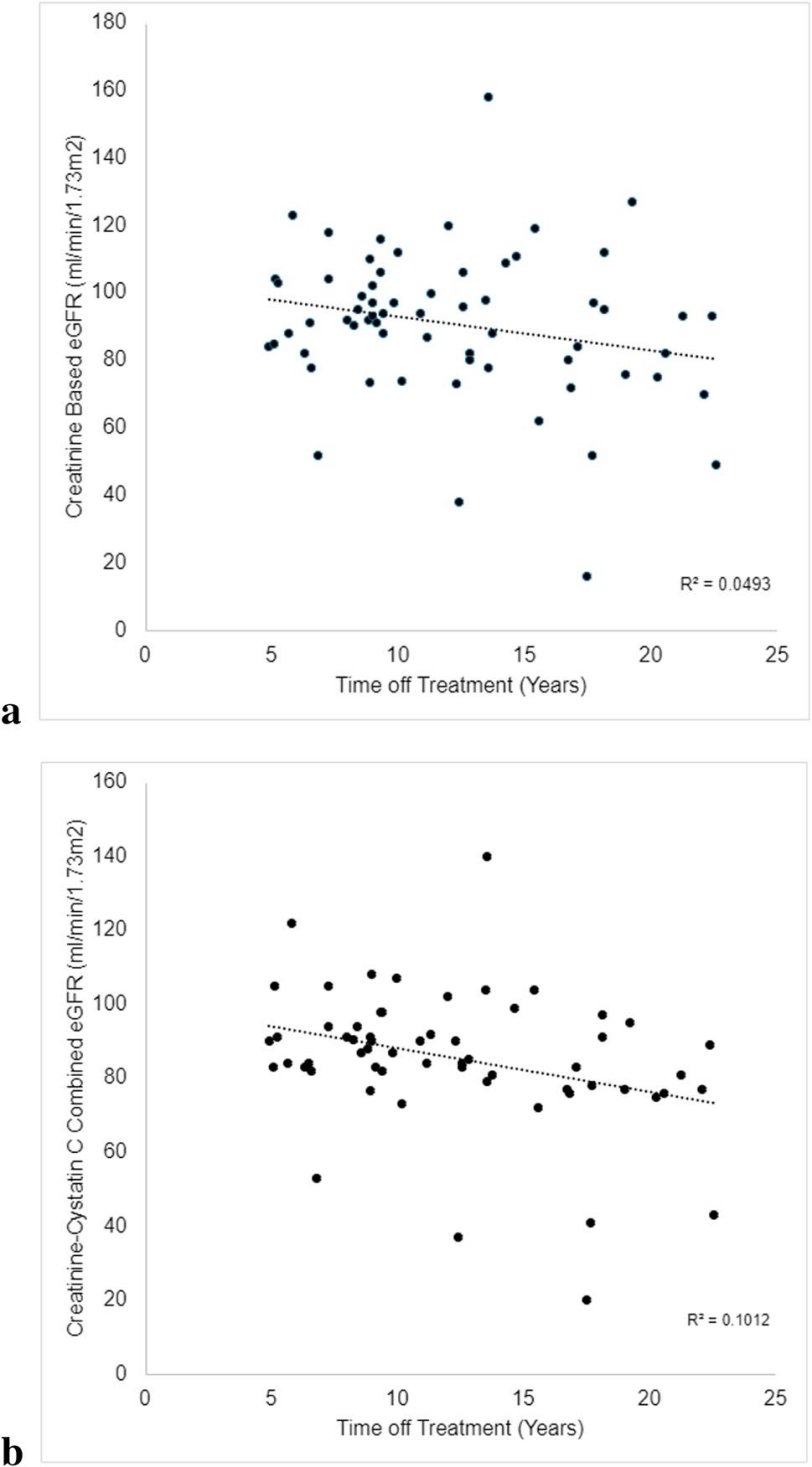
The trend of eGFR with increasing follow-up intervals using **a** creatinine-based eGFR and **b** creatinine-cystatin C–combined eGFR

Patients had a median of 11.25 years off treatment (range 6.5–29.2 years) and were an average of 15.5 years old (range 6.5–30 years old). A serum creatinine was available for all 64 patients, serum cysC was available for 60 patients. Thirty-four patients had a decreased eGFR (< 90 mL/min/1.73 m^2^) based on U25-combined eGFR (Table 2). An additional patient did not have a cysC available but had decreased eGFR based on serum creatinine alone. Eleven additional patients had decreased eGFR when using cystatin C–only eGFR. Five patients had a combined eGFR < 60 mL/min/1.73 m^2^, including the patient who had a bilateral nephrectomy leading to kidney transplantation, and another patient who was dialysis-dependent at the time of analysis. An additional patient had eGFR < 60 ml/min/1.73 m^2^ when using Cystatin C–only eGFR. When comparing the proportions of patients in each eGFR category using the different estimations, the creatinine-only and cystatin C–only calculations were significantly different (*p* < 0.01), but the other comparisons were not significant. There were no statistically significant differences in clinical or demographic characteristics between patients with eGFR < 90 mL/min/1.73 m^2^ and > 90 mL/min/1.73 m^2^ (Table 1). eGFR decreased with increased follow-up time (Fig. 1). There was no relationship between eGFR and age at diagnosis or whole abdominal radiation therapy (WART) (Table 3). Fifty percent of African American or Hispanic patients had a decreased eGFR (Supplemental Table S1). Three of the 5 patients with eGFR < 60 mL/min/1.73 m^2^ were African American. Sixteen of the patients with eGFR < 90 mL/min/1.73 m^2^ had never been seen by nephrology.

**Table 2:** CKiD U25 eGFR

eGFR (ml/min/1.73 m^2^)	> 90	60–90	< 60
Creatinine only	37 (58%)	22 (34%)	5 (8%)
Cystatin C only	14 (23%)	40 (66%)	6 (15%)
Creatinine-cystatin C–combined	26 (43%)	29 (48%)	5 (8%)

*eGFR*, estimated glomerular filtration rate

**Table 3:** Effect of whole abdominal radiation on kidney outcomes

	Whole abdominal radiation	No abdominal radiation	*P* value
BP at most recent visit			0.21
Normal	6 (55%)	32 (60%)	
Elevated	5 (45%)	13 (25%)	
Hypertension	0	8 (15%)	
Compensatory hypertrophy			0.08
Yes	5 (45%)	35 (73%)	
No	6 (55%)	13 (27%)	
eGFR			0.35
≥ 90	6 (55%)	31 (58%)	
60–90	3 (27%)	19 (36%)	
< 60	2 (18%)	3 (6%)	

Data are shown as n (%); *BP*, blood pressure; *eGFR*, estimated glomerular filtration rate

Creatinine-cystatin C–combined eGFR used when available, otherwise based on creatinine-only eGFR

### Proteinuria

Thirty-seven patients were evaluated for proteinuria with a random UPC ratio. Eight of the patients (22%) were found to have proteinuria. Of those 8 patients, 3 had resolution of proteinuria on subsequent evaluations, 3 patients had persistent proteinuria, and 2 patients did not have a follow-up UPC ratio. Two patients received treatment by nephrology for proteinuria.

### Compensatory hypertrophy

Five patients were not included in the CH results: 1 did not have kidney ultrasound results in the electronic health record, 3 underwent only a partial nephrectomy, as opposed to a radical nephrectomy, and 1 underwent a kidney transplant. Of the remaining 59 patients, 67% had CH in their contralateral kidney (Supplemental Table S2). There was no relationship between CH and age at diagnosis or WART (Table 3). Patients who self-identified as African American or Hispanic had CH 45% of the time (Supplemental Table S1).

### Hypertension

Twenty-six patients (41%) had an abnormal BP on at least 2 of the last 3 clinic measurements, with the highest BP being elevated (18 patients), stage 1 HTN (7 patients), or stage 2 HTN (1 patient). Nine patients had an ABPM and 3 were found to have hypertension, one of which was masked (one with INH, two with sustained HTN). In addition, 4 of the patients without hypertension on ABPM were found to have a non-dipping pattern. Two patients were being treated with an antihypertensive medication. Patients had a median of 11.3 years off treatment (range 5–22.6 years) and were an average of 16.7 years old (range 6.5–30 years old). The majority of patients (42 patients) had a normal BMI, while 7 patients were overweight, 13 patients were obese, and 2 patients were underweight at the time of their last visit. Neither age at diagnosis nor WART (Table 3) affected BP outcomes. Five out of twelve (42%) patients who self-identified as African American or Hispanic had abnormal BP (Supplemental Table S1). Twelve of the patients with abnormal BP (46%) had not been seen by nephrology.

### Risk of late-onset kidney failure

Using the CCSS model, all patients had either a moderate (52 patients) or high (12 patients) risk of kidney failure by age 40 (Supplemental Table S3). Of the 5 patients with eGFR < 60 ml/min/1.73 m^2^, 3 had been categorized as having a high risk of late-onset kidney failure (7.5% probability, 29.5 RR compared with siblings).

## Discussion

In this retrospective study, we assessed long-term kidney outcomes in S-WT, including those at high risk for negative outcomes due to WART, nephrotoxic chemotherapy, and bilateral disease. Given that the survival rate following treatment for WT has dramatically improved in recent years, it is important to assess long-term kidney outcomes in this population. Several prior studies have evaluated kidney function outcomes in S-WT and concluded that the risk for significant long-term kidney dysfunction is low. Breslow et al. found the risk of CKD stage 5 20 years from diagnosis to be less than 1% in non-syndromic unilateral WT (UWT) [26]. Similarly, Interiano et al. found that no patients with UWT treated without nephrotoxic chemotherapy or ionizing radiation had eGFR < 60 ml/min/1.73 m^2^ [15]. They therefore concluded that this population was at low risk of significant long-term kidney dysfunction, even though 21% of patients had an eGFR < 90 ml/min/1.73 m^2^. Bailey et al. also found that 23% of their study population had mildly reduced GFR (< 90 ml/min/1.73 m^2^) and concluded that the magnitude of these abnormalities was small and unlikely to result in clinical sequelae [4]. Ages at the time of follow-up for these studies are not reported, but based on the age at diagnosis and follow-up duration, it can be extrapolated that the majority of these patients were still adolescents and young adults at the time of kidney function measurement. Arslan et al. reported their patients were a median of 12 years at the time of follow-up with 75% being less than 20 years of age, and 39% of patients already had decreased eGFR [17].

We found similar results in our study population, with only 2 patients having CKD stage 5 at follow-up. One of these patients presented with bilateral disease at diagnosis leading to bilateral nephrectomy and kidney transplantation. The other received both WART and carboplatin. However, in addition to these two patients, over half had evidence of decreased GFR. This is of particular importance, given that a decreasing trend in GFR was seen with increased follow-up time. The decline in GFR is a normal physiologic process, but a GFR below 90 ml/min/1.73 m^2^ typically does not occur until late adulthood in healthy individuals [27]. Kostel et al. also saw a trend of decreasing GFR with longer follow-up intervals and calculated that patients would have progressed to CKD after 50 years [14]. In fact, one longitudinal study evaluated kidney function in patients up to five decades after surgery for unilateral kidney tumor and found that there was a significant progressive decrease in eGFR from the 3rd to the 5th decade, more than expected for the physiologic kidney function decline typically seen with aging [28]. The patients in our study were on average 15.5 years old, with the oldest patient being 30 years old, and are at risk for further CKD progression throughout their lives. Using the CCSS prediction model for CKD stage 5, 12 patients in our analysis were classified as at high risk of CKD stage 5 by age 40. Twenty-five percent of these patients had already progressed to CKD stage 3 or later by the time of analysis.

The effect of WART on the function of the remaining kidney remains unclear. Our study found no difference in GFR, incidence of hypertension, or CH in patients whose treatment included WART, when compared to those with no radiation affecting the remaining kidney. De Graaf reported that children who received radiation to a field including the remaining kidney had decreased GFR compared with those who had no radiation to the remaining kidney [29]. However, the follow-up time was quite short, with GFR being evaluated only 1 year after nephrectomy. Other studies with longer follow-up times have not found radiation therapy to result in kidney dysfunction [4, 14]; however, this may be due to the small number of patients receiving radiation to the remaining kidney in these studies (five and eight). The CCSS also reported that radiation to the contralateral kidney did not increase the risk of kidney failure but cited too few cases of kidney failure in this cohort as a possible reason [30]. In a study by Green et al., GFR calculated by DTPA plasma clearance was significantly lower among patients treated with WART, compared with unirradiated S-WT. However, the mean was still > 75 mL/min/1.73 m^2^, so it was concluded that this effect of WART was not clinically significant [31]. In addition to decreased eGFR, some studies have also reported an association between WART and proteinuria or lack of CH [32], while others have reported no significant difference in rates of CH [33, 34] or hypertension [30, 31] in irradiated patients. Seven (64%) of the patients who received WART in our study were not assessed for proteinuria, so we were unable to assess for correlation between WART and proteinuria in this study. Given that most patients being treated for WT do not receive WART, a larger multi-center study may be necessary to fully delineate the effects of radiation on kidney function.

Several studies have reported significant rates of proteinuria in S-WT [4, 16, 17]. Arslan et al. specifically evaluated for albuminuria (glomerular proteinuria), which was found in 23% of S-WT. The assessment of proteinuria in this study was impeded by the fact that a large portion of the study was not evaluated for proteinuria. Also, some survivors found to have renal protein loss did not have proteinuria reassessed to see if it was transient or persistent. This is an important finding, given that proteinuria is not only a major and early prognostic indicator of CKD progression, but is also a modifiable clinical risk factor [35–37]. Both elevated and nephrotic range proteinuria are predictors of more rapid CKD progression in children [36]. However, reduction in proteinuria has been shown to be a significant independent predictor of delayed progression of kidney disease [37]. Given the small number of patients adequately assessed for proteinuria, we were not able to evaluate for correlation between treatment therapies and proteinuria.

Previous studies have reported varying rates of CH in S-WT, ranging from 55 to 95% [4, 14, 32]. Our study demonstrated CH in 67% of patients, in line with prior literature. Multiple studies have found an association between younger age at the time of nephrectomy and impaired CH; however, poor growth has not consistently been associated with lower eGFR [14, 32]. No relationship between age at the time of nephrectomy and CH was seen in our study. Additionally, the presence of CH did not appear to affect eGFR.

Despite some smaller initial studies reporting no or minimal increased rates of hypertension in S-WT compared with the general population [4, 38], more recent studies have used ABPM to demonstrate that S-WT have increased BP compared with healthy matched controls, with HTN in 20–30% of survivors, even in early adulthood [17, 31, 39]. One study found an abnormal ABPM in 76% of survivors, with 34% having masked hypertension [40]. This is consistent with rates of masked hypertension in children with CKD stages 2–4 [41]. ABPM is therefore recommended by the recent AAP Clinical Practice Guidelines for children with CKD [42]. Although most patients in our study had normal or elevated BP at the most recent follow-up, only 9 patients had an ABPM. Thirty-three percent of patients with an ABPM were found to have hypertension, including one patient who had a normal clinic BP. It has been suggested that radiation therapy and anthracycline-containing chemotherapy regimens are associated with higher BP; however, the small sample size of this study did not allow us to evaluate the effect of radiation therapy or anthracyclines on blood pressure. Given that hypertension is a modifiable risk factor for CKD progression [43, 44], WT survivors should have their BP monitored closely, with ABPMs obtained if there is any concern for abnormal BP or CKD progression.

Disparities in cancer burden and access to cancer care are well described in adults [45]. In recent years, studies have also begun to evaluate the impact of racial/ethnic and socioeconomic factors on outcomes in childhood cancers. Multiple studies have reported that Black children are more likely to present with more advanced-stage WT, when compared with White children [46, 47]. Similarly, when looking at outcomes for children with non-CNS solid tumor malignancies (including WT), Austin et al. not only found that children with advanced-stage disease at diagnosis were more likely to be Hispanic or non-Hispanic Black children, but Hispanic and non-Hispanic Black children in the lowest SES quartile had the worst 1- and 5-year overall survival [45]. Although the small sample size in our study does not allow us to report any statistically significant differences in the outcomes of racial/ethnic minorities, African American and Hispanic children in our study tended to present at a later stage and appeared to have worse long-term kidney outcomes. Further research is needed on this topic.

Our study has some notable limitations. Our sample size is small, which limited statistical power and prevented stratifying analyses to evaluate the impact of specific chemotherapy regimens. A limitation regarding the evaluation of BP is the fact that only a small number of S-WT had an ABPM. Additionally, we had no internal control group to compare outcomes. A strength of our study is the fact that we included all S-WT, including some who would have been excluded in prior studies, such as those with bilateral disease or treatment including radiation or nephrotoxic chemotherapy. The long duration of follow-up is another strength of this study.

In conclusion, although CKD stage 5 was rare in this study, it should be noted that these patients were still very young (6–30 years old) and were at risk for progression of kidney disease over time. This population appears to be at higher risk of proteinuria, HTN, and decreased kidney function and therefore should be monitored carefully for the development of adverse kidney outcomes as part of their routine survivorship care. Patients should be educated that kidney sequelae can appear decades after they finish treatment, and monitoring of their kidney health should continue throughout their lifetimes. Larger, multi-centered studies are needed to assess the effect of nephrotoxic chemotherapy, WART, and age at diagnosis on long-term kidney outcomes. Additional studies are needed to assess the impact of race, ethnicity, and socioeconomic status on kidney outcomes in S-WT.

## Supplementary information

Below is the link to the electronic supplementary material.

Graphical abstract (PPTX 99.1 KB)

Supplementary file2 (DOCX 24 KB)

## Data Availability

The datasets generated and analyzed during the current study are available from the corresponding author on reasonable request.

## References

[1] Szychot E, Apps J, Pritchard-Jones K (2014) Wilms’ tumor: biology, diagnosis and treatment. Transl Pediatr 3:12–24. 10.3978/j.issn.2224-4336.2014.01.09 26835318 10.3978/j.issn.2224-4336.2014.01.09 PMC4728859

[2] Lopes RI, Lorenzo A (2017) Recent advances in the management of Wilms’ tumor. F1000Res 6:670. 10.12688/f1000research.10760.1 10.12688/f1000research.10760.1 PMC5461897 28620463

[3] Metzger ML, Dome JS (2005) Current therapy for Wilms’ tumor. Oncologist 10:815–826. 10.1634/theoncologist.10-10-815 16314292 10.1634/theoncologist.10-10-815

[4] Bailey S, Roberts A, Brock C, Price L, Craft AW, Kilkarni R, Lee RE, Skillen AW, Skinner R (2002) Nephrotoxicity in survivors of Wilms’ tumours in the North of England. Br J Cancer 87:1092–1098. 10.1038/sj.bjc.6600608 12402147 10.1038/sj.bjc.6600608 PMC2376198

[5] Filler G, Huang SH, Yasin A (2012) The usefulness of cystatin C and related formulae in pediatrics. Clin Chem Lab Med 50:2081–2091. 10.1515/cclm-2012-0257 23093265 10.1515/cclm-2012-0257

[6] Meacham LR, Gurney JG, Mertens AC, Ness KK, Sklar CA, Robison LL, Oeffinger KC (2005) Body mass index in long-term adult survivors of childhood cancer: a report of the Childhood Cancer Survivor Study. Cancer 103:1730–1739. 10.1002/cncr.20960 15761876 10.1002/cncr.20960

[7] Wilson CL, Liu W, Chemaitilly W, Howell CR, Srivastava DK, Howell RM, Hudson MM, Robison LL, Ness KK (2020) Body composition, metabolic health, and functional impairment among adults treated for abdominal and pelvic tumors during childhood. Cancer Epidemiol Biomarkers Prev 29:1750–1758. 10.1158/1055-9965.EPI-19-1321 32796078 10.1158/1055-9965.EPI-19-1321 PMC7721344

[8] de Oliveira WE, Murra MS, Tufi LMB, Cavalcante CEB, de Oliveira MA, da Costa RFA, Rosa BR, da Silva RZM, Ribeiro RC, Ladas EJ, Barr RD (2023) Sarcopenia in children with Wilms tumor: a marker of undernutrition which may impact adversely on clinical outcomes. J Pediatr Hematol Oncol 45:e817–e822. 10.1097/MPH.0000000000002732 37526408 10.1097/MPH.0000000000002732

[9] Ylinen EA, Ala-Houhala M, Harmoinen AP, Knip M (1999) Cystatin C as a marker for glomerular filtration rate in pediatric patients. Pediatr Nephrol 13:506–509. 10.1007/s004670050647 10452279 10.1007/s004670050647

[10] Dharnidharka VR, Kwon C, Stevens G (2002) Serum cystatin C is superior to serum creatinine as a marker of kidney function: a meta-analysis. Am J Kidney Dis 40:221–226. 10.1053/ajkd.2002.34487 12148093 10.1053/ajkd.2002.34487

[11] Pierce CB, Muñoz A, Ng DK, Warady BA, Furth SL, Schwartz GJ (2021) Age- and sex-dependent clinical equations to estimate glomerular filtration rates in children and young adults with chronic kidney disease. Kidney Int 99:948–956. 10.1016/j.kint.2020.10.047 33301749 10.1016/j.kint.2020.10.047 PMC9083470

[12] Ng DK, Pierce CB (2021) Kidney disease progression in children and young adults with pediatric CKD: epidemiologic perspectives and clinical applications. Semin Nephrol 41:405–415. 10.1016/j.semnephrol.2021.09.002 34916001 10.1016/j.semnephrol.2021.09.002 PMC8694646

[13] Finklestein JZ, Norkool P, Green DM, Breslow N, D’Angio GJ (1993) Diastolic hypertension in Wilms’ tumor survivors: a late effect of treatment? A report from the National Wilms’ Tumor Study Group. Am J Clin Oncol 16:201–205 8393271

[14] Kostel Bal AS, Yalcin B, Susam-Şen H, Aydin B, Varan A, Kutluk T, Akyüz C (2016) Renal late effects after the treatment of unilateral nonsyndromic Wilms tumor. J Pediatr Hematol Oncol 38:e147-150. 10.1097/MPH.0000000000000557 26989912 10.1097/MPH.0000000000000557

[15] Interiano RB, Delos Santos N, Huang S, Srivastava DK, Robison LL, Hudson MM, Green DM, Davidoff AM (2015) Renal function in survivors of nonsyndromic Wilms tumor treated with unilateral radical nephrectomy. Cancer 121:2449–2456. 10.1002/cncr.29373 25832759 10.1002/cncr.29373 PMC5161342

[16] Green DM (2013) Evaluation of renal function after successful treatment for unilateral, non-syndromic Wilms tumor. Pediatr Blood Cancer 60:1929–1935. 10.1002/pbc.24738 24039069 10.1002/pbc.24738

[17] Arslan E, Saygili S, Celkan TT, Kurugoglu S, Elicevik M, Camcioglu AE, Konukoglu D, Apak H, Caliskan S, Sever L, Canpolat N (2022) Increased risk for kidney sequelae surrogates in survivors of Wilms tumor. Pediatr Nephrol 37:2415–2426. 10.1007/s00467-022-05460-1 35118543 10.1007/s00467-022-05460-1

[18] Schwartz GJ, Cox C, Seegmiller JC, Maier PS, DiManno D, Furth SL, Warady BA, Munoz A (2020) Recalibration of cystatin C using standardized material in Siemens nephelometers. Pediatr Nephrol 35:279–285. 10.1007/s00467-019-04389-2 31680199 10.1007/s00467-019-04389-2 PMC7249730

[19] Benoit SW, Kathman T, Patel J, Stegman M, Cobb C, Hoehn J, Devarajan P, Nehus EJ (2021) GFR estimation after cystatin C reference material change. Kidney Int Rep 6:429–436. 10.1016/j.ekir.2020.11.028 33615068 10.1016/j.ekir.2020.11.028 PMC7879112

[20] Obrycki Ł, Sarnecki J, Lichosik M, Sopińska M, Placzyńska M, Stańczyk M, Mirecka J, Wasilewska A, Michalski M, Lewandowska W, Dereziński T, Pac M, Szwarc N, Annusewicz K, Rekuta V, Ažukaitis K, Čekuolis A, Wierzbicka-Rucińska A, Jankauskiene A, Kalicki B, Jobs K, Tkaczyk M, Feber J, Litwin M (2022) Kidney length normative values in children aged 0–19 years - a multicenter study. Pediatr Nephrol 37:1075–1085. 10.1007/s00467-021-05303-5 34657197 10.1007/s00467-021-05303-5 PMC9023417

[21] Wu NL, Chen Y, Dieffenbach BV, Ehrhardt MJ, Hingorani S, Howell RM, Jefferies JL, Mulrooney DA, Oeffinger KC, Robison LL, Weil BR, Yuan Y, Yasui Y, Hudson MM, Leisenring WM, Armstrong GT, Chow EJ (2023) Development and validation of a prediction model for kidney failure in long-term survivors of childhood cancer. J Clin Oncol 41:2258–2268. 10.1200/JCO.22.01926 36795981 10.1200/JCO.22.01926 PMC10448933

[22] Farmakis SG, Barnes AM, Carey JC, Braddock SR (2019) Solid tumor screening recommendations in trisomy 18. Am J Med Genet A 179:455–466. 10.1002/ajmg.a.61029 30637956 10.1002/ajmg.a.61029

[23] Clericuzio CL, Martin RA (2009) Diagnostic criteria and tumor screening for individuals with isolated hemihyperplasia. Genet Med 11:220–222. 10.1097/GIM.0b013e31819436cf 19367194 10.1097/GIM.0b013e31819436cf PMC3111026

[24] Mahamdallie SS, Hanks S, Karlin KL, Zachariou A, Perdeaux ER, Ruark E, Shaw CA, Renwick A, Ramsay E, Yost S, Elliott A, Birch J, Capra M, Gray J, Hale J, Kingston J, Levitt G, McLean T, Sheridan E, Seal S, Stiller C, Sebire N, Westbrook TF, Rahman N (2015) Mutations in the transcriptional repressor REST predispose to Wilms tumor. Nat Genet 47:1471–1474. 10.1038/ng.3440 26551668 10.1038/ng.3440

[25] DeBaun MR, Tucker MA (1998) Risk of cancer during the first four years of life in children from The Beckwith-Wiedemann Syndrome Registry. J Pediatr 132:398–400. 10.1016/s0022-3476(98)70008-3 9544889 10.1016/s0022-3476(98)70008-3

[26] Breslow NE, Collins AJ, Ritchey ML, Grigoriev YA, Peterson SM, Green DM (2005) End stage renal disease in patients with Wilms tumor: results from the National Wilms Tumor Study Group and the United States Renal Data System. J Urol 174:1972–1975. 10.1097/01.ju.0000176800.00994.3a 16217371 10.1097/01.ju.0000176800.00994.3a PMC1483840

[27] Hoang K, Tan JC, Derby G, Blouch KL, Masek M, Ma I, Lemley KV, Myers BD (2003) Determinants of glomerular hypofiltration in aging humans. Kidney Int 64:1417–1424. 10.1046/j.1523-1755.2003.00207.x 12969161 10.1046/j.1523-1755.2003.00207.x

[28] Cozzi DA, Ceccanti S, Frediani S, Mele E, Cozzi F (2013) Renal function adaptation up to the fifth decade after treatment of children with unilateral renal tumor: a cross-sectional and longitudinal study. Pediatr Blood Cancer 60:1534–1538. 10.1002/pbc.24545 23606234 10.1002/pbc.24545

[29] de Graaf SS, van Gent H, Reitsma-Bierens WC, van Luyk WH, Dolsma WV, Postma A (1996) Renal function after unilateral nephrectomy for Wilms’ tumour: the influence of radiation therapy. Eur J Cancer 32A:465–469. 10.1016/0959-8049(95)00618-4 8814694 10.1016/0959-8049(95)00618-4

[30] Termuhlen AM, Tersak JM, Liu Q, Yasui Y, Stovall M, Weathers R, Deutsch M, Sklar CA, Oeffinger KC, Armstrong G, Robison LL, Green DM (2011) Twenty-five year follow-up of childhood Wilms tumor: a report from the Childhood Cancer Survivor Study. Pediatr Blood Cancer 57:1210–1216. 10.1002/pbc.23090 21384541 10.1002/pbc.23090 PMC4634648

[31] Green DM, Wang M, Krasin MJ, Davidoff AM, Srivastava D, Jay DW, Ness KK, Shulkin BL, Spunt SL, Jones DP, Lanctot JQ, Shelton KC, Brennan RC, Mulrooney DA, Ehrhardt MJ, Gibson TM, Kurt BA, Robison LL, Hudson MM (2020) Long-term renal function after treatment for unilateral, nonsyndromic Wilms tumor. A report from the St. Jude Lifetime Cohort Study. Pediatr Blood Cancer 67:e28271. 10.1002/pbc.28271 10.1002/pbc.28271 PMC7735383 32706494

[32] Levitt GA, Yeomans E, Dicks Mireaux C, Breatnach F, Kingston J, Pritchard J (1992) Renal size and function after cure of Wilms’ tumour. Br J Cancer 66:877–882. 10.1038/bjc.1992.378 1329909 10.1038/bjc.1992.378 PMC1977990

[33] Cassady JR, Lebowitz RL, Jaffe N, Hoffman A (1981) Effect of low dose irradiation on renal enlargement in children following nephrectomy for Wilms’ tumor. Acta Radiol Oncol 20:5–8. 10.3109/02841868109130183 6264743 10.3109/02841868109130183

[34] Walker RD, Reid CF, Richard GA, Talbert JL, Rogers BM (1982) Compensatory renal growth and function in postnephrectomized patients with Wilms tumor. Urology 19:127–130. 10.1016/0090-4295(82)90564-7 6277068 10.1016/0090-4295(82)90564-7

[35] Fuhrman DY, Schneider MF, Dell KM, Blydt-Hansen TD, Mak R, Saland JM, Furth SL, Warady BA, Moxey-Mims MM, Schwartz GJ (2017) Albuminuria, proteinuria, and renal disease progression in children with CKD. Clin J Am Soc Nephrol 12:912–920. 10.2215/CJN.11971116 28546440 10.2215/CJN.11971116 PMC5460717

[36] Warady BA, Abraham AG, Schwartz GJ, Wong CS, Muñoz A, Betoko A, Mitsnefes M, Kaskel F, Greenbaum LA, Mak RH, Flynn J, Moxey-Mims MM, Furth S (2015) Predictors of rapid progression of glomerular and nonglomerular kidney disease in children and adolescents: the chronic kidney disease in children (CKiD) cohort. Am J Kidney Dis 65:878–888. 10.1053/j.ajkd.2015.01.008 25799137 10.1053/j.ajkd.2015.01.008 PMC4578873

[37] ESCAPE Trial Group; Wühl E, Trivelli A, Picca S, Litwin M, Peco-Antic A, Zurowska A, Testa S, Jankauskiene A, Emre S, Caldas-Afonso A, Anarat A, Niaudet P, Mir S, Bakkaloglu A, Enke B, Montini G, Wingen AM, Sallay P, Jeck N, Berg U, Caliskan S, Wygoda S, Hohbach-Hohenfellner K, Dusek J, Urasinski T, Arbeiter K, Neuhaus T, Gellermann J, Drozdz D, Fischbach M, Möller K, Wigger M, Peruzzi L, Mehls O, Schaefer F (2009) Strict blood-pressure control and progression of renal failure in children. N Engl J Med 361:1639-1650. 10.1056/NEJMoa0902066 10.1056/NEJMoa0902066 19846849

[38] Kantor AF, Li FP, Janov AJ, Tarbell NJ, Sallan SE (1989) Hypertension in long-term survivors of childhood renal cancers. J Clin Oncol 7:912–915. 10.1200/JCO.1989.7.7.912 2544684 10.1200/JCO.1989.7.7.912

[39] Elli M, Sungur M, Genç G, Ayyildiz P, Dagdemir A, Pinarli FG, Acar S (2013) The late effects of anticancer therapy after childhood Wilm’s tumor: the role of diastolic function and ambulatory blood pressure monitoring. Jpn J Clin Oncol 43:1004–1011. 10.1093/jjco/hyt105 23924525 10.1093/jjco/hyt105

[40] Chu DI, Ehlayel AM, Ginsberg JP, Meyers KE, Benton M, Thomas M, Carlson C, Kolon TF, Tasian GE, Greenberg JH, Furth SL, Denburg MR (2021) Kidney outcomes and hypertension in survivors of Wilms tumor: a prospective cohort study. J Pediatr 230:215-220.e211. 10.1016/j.jpeds.2020.12.005 33290810 10.1016/j.jpeds.2020.12.005 PMC7914174

[41] Mitsnefes M, Flynn J, Cohn S, Samuels J, Blydt-Hansen T, Saland J, Kimball T, Furth S, Warady B; CKiD Study Group (2010) Masked hypertension associates with left ventricular hypertrophy in children with CKD. J Am Soc Nephrol 21:137-144. 10.1681/ASN.2009060609 10.1681/ASN.2009060609 PMC2799282 19917781

[42] Flynn JT, Kaelber DC, Baker-Smith CM, Blowey D, Carroll AE, Daniels SR, de Ferranti SD, Dionne JM, Falkner B, Flinn SK, Gidding SS, Goodwin C, Leu MG, Powers ME, Rea C, Samuels J, Simasek M, Thaker VV, Urbina EM, Subcommittee on Screening and Management of High Blood Pressure in Children, (2017) Clinical practice guideline for screening and management of high blood pressure in children and adolescents. Pediatrics 140:e20173035. 10.1542/peds.2017-1904 28827377 10.1542/peds.2017-1904

[43] Peterson JC, Adler S, Burkart JM, Greene T, Hebert LA, Hunsicker LG, King AJ, Klahr S, Massry SG, Seifter JL (1995) Blood pressure control, proteinuria, and the progression of renal disease. the Modification of Diet in Renal Disease Study. Ann Intern Med 123:754–762. 10.7326/0003-4819-123-10-199511150-00003 7574193 10.7326/0003-4819-123-10-199511150-00003

[44] Wingen AM, Fabian-Bach C, Schaefer F, Mehls O (1997) Randomised multicentre study of a low-protein diet on the progression of chronic renal failure in children. European Study Group of Nutritional Treatment of Chronic Renal Failure in Childhood. Lancet 349:1117–1123. 10.1016/s0140-6736(96)09260-4 9113009 10.1016/s0140-6736(96)09260-4

[45] Austin MT, Nguyen H, Eberth JM, Chang Y, Heczey A, Hughes DP, Lally KP, Elting LS (2015) Health disparities are important determinants of outcome for children with solid tumor malignancies. J Pediatr Surg 50:161–166. 10.1016/j.jpedsurg.2014.10.037 25598116 10.1016/j.jpedsurg.2014.10.037 PMC4408987

[46] Apple AN, Neuzil KE, Phelps HM, Li B, Lovvorn Iii HN (2021) Race disparities in genetic alterations within Wilms tumor specimens. J Pediatr Surg 56:1135–1141. 10.1016/j.jpedsurg.2021.02.030 33745745 10.1016/j.jpedsurg.2021.02.030

[47] Axt J, Murphy AJ, Seeley EH, Martin CA, Taylor C, Pierce J, Caprioli RM, Whiteside M, Lovvorn HN (2011) Race disparities in Wilms tumor incidence and biology. J Surg Res 170:112–119. 10.1016/j.jss.2011.03.011 21529835 10.1016/j.jss.2011.03.011 PMC3150230

