# Supplementary material for: Long-term kidney outcomes in survivors of Wilms tumor: a single-center retrospective cohort study
Source: Pediatr Nephrol. 2025 Jan 9;40(5):1603–11. doi: 10.1007/s00467-024-06624-x (PMC11947031; doi:10.1007/s00467-024-06624-x)
Supplement: Supplementary file 1 — Graphical abstract (PPTX 99.1 KB) [file 467_2024_6624_MOESM1_ESM.pptx]

## Slide 1
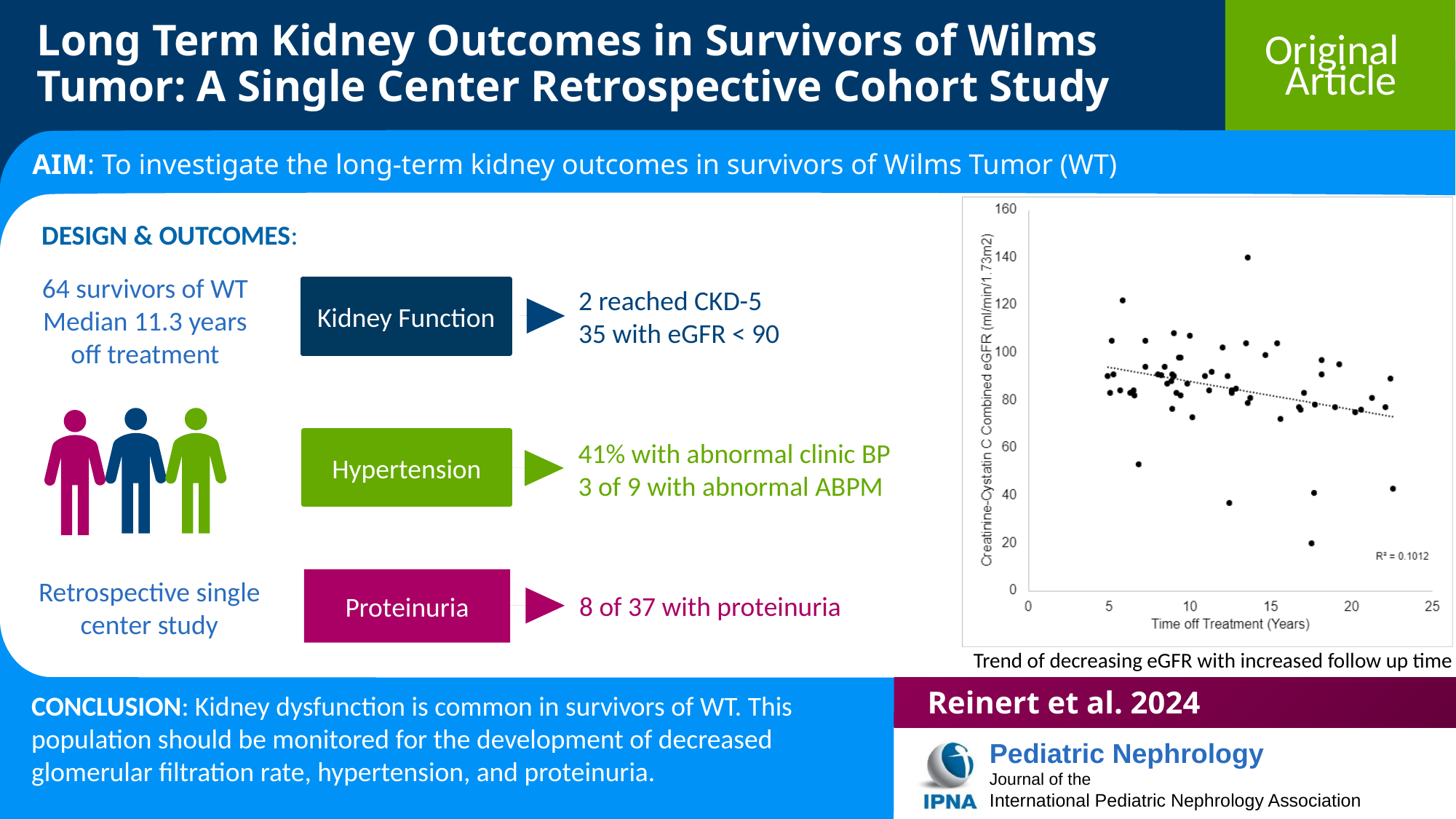

Long Term Kidney Outcomes in Survivors of Wilms Tumor: A Single Center Retrospective Cohort Study
AIM: To investigate the long-term kidney outcomes in survivors of Wilms Tumor (WT)
DESIGN & OUTCOMES:
64 survivors of WT
Median 11.3 years off treatment
2 reached CKD-5
35 with eGFR < 90
Kidney Function
Hypertension
41% with abnormal clinic BP
3 of 9 with abnormal ABPM
Retrospective single center study
Proteinuria
8 of 37 with proteinuria
Trend of decreasing eGFR with increased follow up time
Reinert et al. 2024
CONCLUSION: Kidney dysfunction is common in survivors of WT. This population should be monitored for the development of decreased glomerular filtration rate, hypertension, and proteinuria.
