# Supplementary material for: Long-term kidney outcomes in survivors of Wilms tumor: a single-center retrospective cohort study
Source: Pediatr Nephrol. 2025 Jan 9;40(5):1603–11. doi: 10.1007/s00467-024-06624-x (PMC11947031; doi:10.1007/s00467-024-06624-x)
Supplement: Supplementary file 2 — Supplementary file2 (DOCX 24 KB) [file 467_2024_6624_MOESM2_ESM.docx]

**Long-term kidney outcomes in survivors of Wilms Tumor: a single center retrospective cohort study**

Shannon Reinert (corresponding author), Stefanie Benoit, and Rajaram Nagarajan

Department of Nephrology, Cincinnati Children’s Hospital Medical Center, Cincinnati, OH. Email: Shannon.reinert@cchmc.org

**Table of Contents:**

**Page Contents**

**2 Table S1:** Kidney Outcomes in patients of minority race and ethnicity

**3 Table S2:** Relationship between **c**ompensatory hypertrophy and eGFR

**3 Table S3:** Risk of early onset kidney failure using the CCSS kidney failure model

**Table S1.** Kidney outcomes in patients of minority race and ethnicity

| **Stage at Diagnosis** |  | |
| --- | --- | --- |
| 2 | 1 (8%) | |
| 3 | 5 (42%) | |
| 4 | 4 (33%) | |
| 5 | 2 (17%) | |
| **Compensatory Hypertrophy** |  | |
| Yes | 5 (45%) | |
| No | 6 (55%) | |
| **Proteinuria** |  | |
| Yes | 3 (43%) | |
| No | 4 (57%) | |
| **eGFR (ml/min/1.73m^2^)** | CKiD U25-Creatinine | CKiD U25-Combined |
| ≥90 | 6 (50%) | 4 (40%) |
| 60-90 | 3 (25%) | 3 (30%) |
| <60 | 3 (25%) | 3 (30%) |
| **Blood Pressure** |  | |
| Normal | 6 (50%) | |
| Abnormal, No Antihypertensive | 5 (42%) | |
| On Antihypertensive | 1 (8%) | |

Data are shown as n (%)

*eGFR* estimated glomerular filtration rate

**Table S2.** Relationship between compensatory hypertrophy and eGFR

| Kidney Length >2 SD above normal | 40 (of 60 with unilateral nephrectomy, 67%) |
| --- | --- |
| Median eGFR with CH (IQR) | 92 (82-104) ml/min/1.73m2 |
| Median eGFR without CH (IQR) | 95 (81-103) ml/min/1.73m2 |

Data are shown as median (interquartile range) or as n (%)

*eGFR* estimated glomerular filtration rate, *CH* Compensatory hypertrophy

**Table S3.** Risk of early onset kidney failure using the CCSS kidney failure model

|  | Number of patients | Probability of CKD stage 5 by age 40 | RR compared to siblings | Number of patients with eGFR <60 ml/min/1.73m2 |
| --- | --- | --- | --- | --- |
| Moderate | 52 | 2.1% | 11.2 | 2 (4%) |
| High | 12 | 7.5% | 29.5 | 3 (25%) |

*CCSS* Childhood Cancer Survivor Study, *CKD* Chronic Kidney Disease, *eGFR* estimated glomerular filtration rate
